# Risk factors for gastroesophageal reflux disease: a population-based study

**DOI:** 10.1186/s12876-024-03143-9

**Published:** 2024-02-05

**Authors:** Sepehr Sadafi, Ali Azizi, Yahya Pasdar, Ebrahim Shakiba, Mitra Darbandi

**Affiliations:** 1 Clinical Research Development Center, Imam Reza Hospital, Kermanshah University of Medical Sciences, Kermanshah, Iran; 2 Social Development and Health Promotion Research Center, Kermanshah University of Medical Sciences, Kermanshah, Iran; 3 Department of Community and Family Medicine, School of Medicine, Kermanshah University of Medical Sciences, Kermanshah, Iran; 4 Research Center for Environmental Determinants of Health (RCEDH), Health Institute, Kermanshah University of Medical Sciences, Kermanshah, Iran

**Keywords:** Gastroesophageal reflux disease, Anthropometric indices, Physical activity, Dietary, Smoking

## Abstract

**Background:**

Gastroesophageal reflux disease (GERD) in the long term reduces the quality of life, leading to digestive diseases. The present study aims to determine the risk factors for GERD.

**Method:**

This study was conducted on 9,631 adults aged 35–65 years. The demographic characteristics, behavioral habits, nutritional intake, physical activity, anthropometric indices, and GERD data were extracted from the databank related to the Ravansar non-communicable diseases (RaNCD). Statistical analysis was performed using logistic regression models.

**Results:**

The prevalence of GERD was 10.99% (*n* = 1,058). The GERD was higher among older age and women. After adjusting for age and sex, the odds of GERD among current smokers was 23% higher than non-smokers. Drinking increased odds of GERD (OR: 1.51; 95% CI: 1.13, 1.99). The odds of GERD among depressed individuals were 46% higher than non-depressed. In addition, a significant relationship was observed between the high intake of sweets and desserts with increased GERD (OR: 1.02, 95% CI: 1.01, 1.03). Further, high intake of fiber (OR: 0.98, 95% CI: 0.97, 0.99) and dairy (OR: 0.99, 95% CI: 0.98, 0.99) was related to reducing the odds of GERD. Furthermore, a significant relationship was reported between the waist hip ratio (WHR) and visceral fat area (VFA) with increased odds of GERD. Finally, the physical activity level was inversely related to GERD.

**Conclusion:**

Based on the results, smoking, alcohol, inactivity, high intake of sweets and desserts, low intake of fiber, depression, visceral fat, and obesity are considered as risk factors for GERD. Modifying lifestyle and behavioral habits prevent GERD.

## Background

Gastroesophageal reflux disease (GERD) is regarded as a common disorder of the upper gastrointestinal tract, resulting in returning the stomach contents into the esophagus [1, 2]. Heartburn, dysphagia, belching, hiccups, nausea, and vomiting are among less common symptoms of reflux [2, 3]. Based on the estimations, 13.98% of the adult population worldwide suffers from GERD, and its prevalence ranges from 4.16% in China to 22.40% in Turkey [4, 5].

According to scientific documentation, GERD is considered as a multifactorial disorder. Physiological and lifestyle factors are among the main reasons for GERD. The physiological factors include increased compliance of the esophagus-gastric junction (OGJ), higher pressure gradient across the OGJ, and weakness of the lower esophageal sphincter (LES) [6–8]. Genetic factors and polymorphism play a critical role in the development of reflux [4, 9]. However, modifiable risk factors should be controlled. Based on the studies, lifestyle plays a significant role in creating GERD. Smoking, stress, dietary factors (coffee and spicy foods), obesity, inactivity, alcohol intake, and family history of reflux were identified as risk factors for GERD [10, 11].

Regular and recreational physical activity such as running and swimming reduces the risk of GERD [12, 13]. In addition, individuals who walked after dinner had a lower risk of GERD compared to those who lay down during this time [14]. A meta-analysis study (2020) showed that reflux is significantly more common among smokers than non-smokers [5].

Demographic characteristics play a vital role in reflux, as well. Based on some studies, the prevalence of reflux among women is higher than men [5, 15].

GERD in the long term reduces the quality of life, leading to digestive diseases. GERD can be prevented and controlled by identifying its risk factors. A large number of factors may contribute to the development and progression of this disease. However, identifying modifiable risk factors can greatly help control and prevent reflux. Few studies have been conducted on lifestyle-related risk factors for reflux. The population included adults of western Iran and Kurdish ethnicity; whose lifestyle differs somewhat from other populations in Iran. Therefore, reflux is assessed as an annoying disorder under the influence of lifestyle factors in this special population. This study seeks to determine the risk factors for GERD among Iranian adults.

## Method

### Participants

This cross-sectional study was conducted during 2023 utilizing the data related to the baseline phase of Ravansar non-communicable diseases (RaNCD) cohort study on 10,000 adults aged 35–65 years. RaNCD is regarded as a population-based prospective cohort study and part of the Prospective Epidemiological Research Studies in Iran (PERSIAN), which started in Ravansar in Kermanshah province during 2014 and continues for 15 years [16]. All of the participants were included in the first phase of RaNCD study. Before the analysis, 83 individuals were excluded from the study due to neoplasms, 138 pregnant women and 195 cases provided incomplete information, and the analysis was performed on 9,631 participants.

### Measurements

The GERD group answered yes to two questions from the RaNCD medical questionnaire, resulting in confirming their GERD by RaNCD center physician. The physician asked the group members whether they had ever been diagnosed with reflux and whether they experienced reflux of food from the stomach to the esophagus or not [17]. Food regurgitation frequency is divided into four groups including almost daily, several times each week, several times each month, and every once in a while.

Age, sex, place of residence, and education level were extracted applying a personal information questionnaire. In addition, alcohol drinking, smoking, and sleep duration in 24 h were extracted employing behavioral habits questionnaire. Participants who did not smoke regularly or occasionally during the past year and smoked more than 100 cigarettes in their lifetime were considered as former smokers. Current smokers who smoked at least 100 cigarettes a year were classified into three groups including light smoker (1–9 cigarettes per day), moderate smoker (10–19 cigarettes per day), and heavy smoker (20 or more cigarettes per day) based on smoking intensity [18]. Socio-economic status (SES) based on wealth score, residence in city/rural, occupation, and education of the participants was constructed by principal component analysis (PCA) method. The nutritional intake was obtained based on the food frequency questionnaire (FFQ) [19]. Physical activity was evaluated using the Persian Cohort Standard Questionnaire and reported based on MET/hours per day [20]. The anthropometric indices including body mass index (BMI), percent body fat (PBF), body fat mass (BFM), waist hip ratio (WHR), and visceral fat area (VAI) were measured utilizing an Impedance Analyzer BIA (Inbody 770, Korea).

The participants were examined for symptomatic or asymptomatic depression by a psychologist. A clinical examination and a self-administered questionnaire formed the screening process of depression in the studied population. In addition, participants were asked whether they had previously received antidepressants. Metabolic syndrome (MetS) was defined based on the International Diabetes Federation (IDF) criteria [21].

### Statistical analysis

Stata statistical software version 14.2 (Stata Corp, College Station, TX, USA) was used for analysis. Descriptive results are reported in terms of quantity and quality of the variable, with mean ± standard deviation and frequency (percentage), respectively. The difference between the basic characteristics and lifestyle of the two groups with and without GERD has been evaluated with t-test and chi-square tests. The association between GERD and risk factors was performed with univariate and multivariate logistic regression models and reported as odds ratio (OR) and 95% confidence interval (CI). All presented ***P*** values were two-sided, and *p* < 0.05 was considered statistically significant.

## Results

The participants included 9,631 adults aged 35–65 years. The prevalence of GERD was 10.99% (*n* = 1,058). The GERD was higher among older age and women. The prevalence of GERD among participants with high physical activity level was higher than those with low physical activity (18.15 vs. 34.12%). Current and former smokers were more in the group with GERD than the non-GERD group (*P* = 0.036). Alcohol consumption was 13.83 and 10.84% in the group with and without GERD respectively (*P* = 0.043). The food regurgitation was significantly more in participants with GERD than the non-GERD group (*P* < 0.001) (Table 1).

**Table 1:** Basic characteristics and behavioral habits of study participants (*n* = 9,631)

Characteristics	Non- Gastroesophageal reflux	Gastroesophageal reflux	***P*** value
**Number (%)**	8573 (89.01)	1058 (10.99)	
**Age (year)**	47.21 ± 8.25	48.16 ± 8.19	0.004
**Sex**			
Men	4147 (89.66)	478 (10.34)	0.050
Women	4426 (88.41)	580 (11.59)	
**Socioeconomic status**			
Weak	2851 (89.80)	324 (10.20)	0.215
Moderate	2860 (88.76)	362 (11.24)	
Good	2862 (88.50)	372 (11.50)	
**Physical activity (MET hour per day)**			
Low	2549 (29.73)	361 (34.12)	< 0.001
Moderate	4063 (47.39)	505 (47.73)	
High	1961 (22.87)	192 (18.15)	
**Smoking status**			
Current	993 (11.62)	127 (12.08)	0.036
Former	726 (8.50)	115 (10.94)	
Passive	3294 (38.56)	406 (38.63)	
Never	3529 (41.31)	403 (38.34)	
**Smoking intensity current**			
Light	298 (6.59)	39 (7.36)	0.409
Moderate	178 (3.94)	17 (3.21)	
Heavy	517 (11.43)	71 (13.40)	
**Drinking alcohol**	993 (10.84)	65 (13.83)	0.043
**Frequency of regurgitation**			
No	7717 (90.01)	152 (14.36)	< 0.001
Almost daily	47 (0.54)	81 (7.65)	
Several times each week	53 (0.61)	94 (8.88)	
Several times each month	82 (0.95)	135 (12.75)	
Every once in a while	674 (7.86)	596 (56.33)	
**Sleep habit**			
Sleep duration (h/24 h)	7.100 ± 1.22	6.99 ± 1.26	0.005
Daytime napping (minute)	66.31 ± 46.03	65.35 ± 45.56	0.868
**Comorbidity**			
Depression	1011 (10.84)	47 (15.51)	0.010
Metabolic syndrome	668 (10.45)	390 (12.04)	0.018

Descriptive report: Frequency (percentage) for qualitative variables, mean ± Standard deviation for quantitative data

* Using t-test and chi-square test

The VFA was significantly higher in participants with GERD than in those without GERD (12.60 VS. 126.01 cm^2^, *P* = 0.008). The PBF was significantly higher in individuals with GERD than non-GERD group (*P* = 0.003). The average intake of red and white meat in participants with GERD was significantly higher than those without GERD (139.80 vs. 141.12 gr/d, *P* = 0.045). The average intake of dairy products was significantly lower in participants without GERD (*P* = 0.035). The fiber intake was 25.76 ± 0.33 gr/d and 26.75 ± 0.13 gr/d in the GERD and non-GERD group, respectively (*P* = 0.10). Sugar consumption in participants with GERD was significantly higher than the non-GERD group (143.83 ± 68.48 vs. 149.91 ± 62.61, *P* = 0.007). The prevalence of depression (10.84 VS. 15.51%, *P* = 0.010) and metabolic syndrome (10.45 VS. 12.04%, *P* = 0.018) in participants with GERD was significantly higher than the non-GERD group (Table 2).

**Table 2:** Anthropometric indicators and nutritional intake of the participants (*n* = 9,631)

Characteristics	Non- Gastroesophageal reflux	Gastroesophageal reflux	***P*** value*
**Anthropometric indicators**			
BMI (kg/m^2^)	27.47 ± 4.64	27.62 ± 4.56	0.292
WHR	0.94 ± 0.06	0.94 ± 0.06	0.138
VFA (cm^2^)	121.60 ± 51.47	126.01 ± 52.10	0.008
PBF	33.69 ± 9.45	34.60 ± 9.68	0.003
BFM (kg)	24.99 ± 9.56	25.60 ± 9.52	0.052
**Nutritional intake**			
Bread and cereals (gr/d)	550.39 ± 1.73	533.70 ± 4.94	0.065
Legumes (gr/d)	35.71 ± 0.32	35.32 ± 0.92	0.690
Dairy (gr/d)	478.58 ± 3.92	453.64 ± 11.19	0.035
Nuts (gr/d)	9.17 ± 0.11	9.81 ± 0.33	0.066
Sweets and desserts(gr/d)	61.46 ± 0.41	63.52 ± 1.17	0.039
Tea and coffee (gr/d)	752.22 ± 5.42	736.10 ± 15.44	0.324
Salt (gr/d)	4.29 ± 0.03	4.38 ± 0.08	0.364
Red &white meat (gr/d)	139.80 ± 0.74	141.12 ± 2.10	0.045
Egg (gr/d)	21.62 ± 0.21	20.71 ± 0.59	0.148
Vegetables (gr/d)	287.68 ± 1.80	277.63 ± 5.15	0.045
Fruits (gr/d)	290.57 ± 2.19	298.77 ± 6.23	0.214
Hydrogenated fats (gr/d)	20.58 ± 0.21	20.17 ± 0.59	0.514
Fiber (gr/d)	26.75 ± 0.13	25.76 ± 0.33	0.010
Sugar (gr/d)	143.83 ± 68.48	149.91 ± 62.61	0.007
Added sugar (gr/d)	8.89 ± 1.84	8.92 ± 1.74	0.540
**Macronutrients and Energy**			
Carbohydrate (gr/d)	416.21 ± 153.06	401.21 ± 141.98	0.002
Lipid, fat (gr/d)	79.83 ± 34.21	76.77 ± 33.10	0.006
Protein (gr/d)	92.19 ± 37.66	88.27 ± 19.63	0.001
Energy intake (kcal/d)	2716.27 ± 977.43	2613.59 ± 917.88	0.001

Descriptive report: Frequency (percentage) for qualitative variables, mean ± Standard deviation for quantitative data

* Using t-test and chi-square test

After adjusting for age and sex, the odds of GERD among current smokers was 23% higher than non-smokers (OR: 1.23; 95% CI: 1.02, 1.55). Drinking alcohol was related to increased odds of GERD (OR: 1.32; 95% CI: 1.01, 1.73), which remained significant after adjusting for age and sex (OR: 1.51; 95% CI: 1.13, 1.99). After adjusting the confounding variables, the odds of GERD in moderate and high levels of physical activity was 31 and 29% lower compared to low levels of physical activity, respectively.

In the crude and adjusted model, the odds of GERD in depressed individuals were 51 (95% CI: 1.10, 2.07) and 46% (95% CI: 1.06, 2.01) higher than those without such complication, respectively.

In the crude model, GERD in individuals with metabolic syndrome was higher by 17%, which was not significant after adjustment. The consumption of sweets and desserts increased GERD after adjusting the regression model (OR: 1.02, 95% CI: 1.01, 1.03) (OR: 0.98, 95% CI: 0.97, 0.99). Daily intake of fiber (OR: 0.98, 95% CI: 0.97, 0.99) and dairy products (OR: 0.99, 95% CI: 0.98, 0.99) reduced the odds of GERD significantly. The WHR and VFA increased odds of GERD significantly (Table 3).

**Table 3:** Risk factors of gastroesophageal reflux disease by logistic regression analysis

Variables	Model 1*	Model 2**
	OR (95% CI)	OR (95% CI)
**Smoking**		
Never	References	References
Current	1.12 (0.90, 1.38)	1.23 (1.02, 1.55)
Passive	1.10 (0.93, 1.24)	1.10 (0.93, 1.25)
Former	1.39 (1.11, 1.73)	1.43 (1.13, 1.90)
**Smoking intensity current**		
Light	References	References
Moderate	0.73 (0.40, 1.33)	0.74 (0.41, 1.36)
Heavy	1.06 (0.70, 1.60)	1.10 (0.70, 1.65)
**Drinking alcohol**		
No	References	References
Yes	1.32 (1.01, 1.73)	1.51 (1.13, 1.99)
**Physical activity (MET hour per day)**		
Low	References	References
Moderate	0.88 (0.76, 1.01)	0.87 (0.75, 1.02)
High	0.69 (0.57, 0.83)	0.71 (0.58, 0.85)
**Depression**		
No	References	References
Yes	1.51 (1.10, 2.07)	1.46 (1.06, 2.01)
**Metabolic syndrome**		
No	References	References
Yes	1.17 (1.02, 1.33)	1.11 (0.97, 1.27)
**Nutritional intake**		
Sweets and desserts (gr/d)	0.99 (0.99, 1.01)	1.02 (1.01, 1.03)
Sugar (gr/d)	0.99 (0.98, 1.01)	(0.98, 1.01)
Added sugar (gr/d)	1.01 (0.97, 1.04)	1.01 (0.98, 1.05)
Fiber (gr/d)	0.99 (0.98, 0.99)	0.98 (0.97, 0.99)
Tea and coffee (gr/d)	0.99 (0.99, 1.01)	0.99 (0.99, 1.01)
Dairy (gr/d)	0.99 (0.98, 0.99)	0.99 (0.98, 0.99)
**Sleep duration**	0.93 (0.88, 0.98)	0.93 (0.88, 0.98)
**Anthropometric indicators**		
BMI (kg/m^2^)	1.07 (0.99, 1.011)	1.05 (0.99, 1.10)
WHR	2.15 (1.78, 5.93)	1.94 (1.12, 5.23)
VFA (cm^2^)	1.01 (1.01, 1.03)	1.01 (1.01, 1.01)
PBF	1.01 (1.01, 1.02)	1.01 (0.99, 1.02)
BFM (kg)	1.03 (0.99, 1.05)	1.01 (0.98, 1.01)
**Frequency of regurgitation**		
Every once in a while	References	References
Several times each month	2.43 (1.65, 3.57)	2.42 (1.64, 3.55)
Several times each week	2.90 (1.87, 4.52)	2.85 (1.83, 4.44)
Almost daily	2.72 (1.69, 4.36)	2.66 (1.65, 4.27)

*Unadjusted **Adjusted for age and sex

## Discussion

A cross-sectional analysis of data from 9,631 adults indicated that low physical activity, high intake of sweets and desserts, low intake of fiber, smoking, alcohol consumption, depression, visceral fat, and central obesity are among the most critical risk factors for GERD.

The odds of GERD among current smokers were 23% higher than non-smokers. In addition, drinking increased odds of GERD. Most studies have found a positive relationship between smoking and GERD. A meta-analysis study revealed that the overall prevalence of GERD is higher among current smokers compared to both ex- and non-smokers. However, the OR and relative risk (RR) were found to be insignificant among current smokers to non- and ex-smokers (OR and RR = 1.04, *P* = 0.065) [22]. According to Baklola et al., smokers are at a greater risk of developing GERD compared to non-smokers [10].

Several studies have reported inconsistent results regarding the relationship between alcohol consumption and reflux. In addition, Matsuzaki et al. reported a positive correlation between excessive daily alcohol consumption and the severity of reflux esophagitis and Barrett’s esophagus among Japanese men [23]. The study conducted on 513 adults revealed that alcohol increases the chance of reflux by 93%. However, such relationship was not statistically significant [24]. Further, Nirvan et al. argued that there is no significant difference in the prevalence of reflux between individuals who consume alcohol in low amounts and those who drink moderate to high amounts [22]. Therefore, longitudinal studies should be conducted to investigate the causal relationship between alcohol and GERD.

This study supports previous results regarding the relationship between physical activity and a lower risk of experiencing GERD. The present study indicates that individuals with moderate and high levels of physical activity have a 31 and 29% lower probability of experiencing GERD compared to those with low levels of physical activity, respectively. Based on the previous studies, the effect of physical activity on the occurrence of reflux symptoms is related to the type of exercise, activity level, and its duration [25, 26]. For example, Djarv et al. claimed that physical activity can lower the risk of GERD among obese individuals. However, no significant correlation was reported for non-obese individuals [27]. Some asserted that physical activity and intense exercise after eating can prevent GERD symptoms in healthy individuals and athletes [14, 28]. Individuals who engage in activities such as weightlifting or carrying heavy objects report a decrease in symptoms of acid reflux compared to those leading a sedentary lifestyle [29]. Increasing physical activity levels is highly recommended as an effective preventive measure against reflux.

A positive relationship was observed between consuming a large amount of sweets and desserts with an elevated risk of experiencing GERD. Conversely, a high intake of fiber and dairy products reduced odd of developing GERD. Based on a review study, fatty, fried, sour, spicy foods, orange and grapefruit juice, tomatoes and canned tomatoes, chocolate, coffee/tea, carbonated beverages, and alcohol are considered as the main triggers for GERD symptoms [30]. Further, Jarosz et al. asserted that the severity of GERD symptoms increased after consuming fatty, fried, sour or spicy foods, as well as sweets. Furthermore, certain factors such as the frequency of consuming small and large meals, daily consumption of mint tea, and indulging in a substantial dinner were identified as risk factors for experiencing GERD [24]. Adhering to a healthy diet reduces the symptoms related to reflux.

The results of our study demonstrate that individuals with GERD have a shorter sleep duration. Similarly, a longitudinal study in Sweden revealed that inadequate and/or short sleep independently raised the risk of nocturnal gastroesophageal reflux during a 10-year follow-up. Hence, insufficient sleep has been proposed as a potential contributor to reflux [31]. Fujiwara et al.‘s study shows a two-way association between sleep duration and reflux [32]. According to Kurin et al. study, there is a two-way connection between GERD and sleep, with GERD being linked to various sleep disorders. Insufficient sleep can worsen GERD, and there is a relationship between nighttime GERD and extraesophageal symptoms. Interestingly, addressing GERD can improve sleep quality, and improving sleep can alleviate GERD symptoms [33]. Research indicates that the physiological alterations during sleep elevate the likelihood of nocturnal reflux in individuals with GERD. Various physiological changes occur during sleep, such as delayed gastric emptying, reduced frequency of transient lower esophageal sphincter relaxation (TLESR), lowered basal upper esophageal sphincter pressure, and alterations in primary and secondary esophageal peristalsis. These sleep-related changes impacting GERD, alongside those affecting the gastrointestinal system, involve decreased salivation and swallowing responses [34–38].

The results represented that the WHR and VFA increased odds of GERD. In another study, Mehta et al. focused on 42, 955 women aged 42–62, declaring that obesity is regarded as a risk factor for reflux [39]. A study on Korean adults identified abdominal obesity as an independent risk factor for erosive esophagitis [40]. Abdominal obesity increases intra-gastric pressure, as well as disrupting the gastroesophageal junction. Hormonal secretion affecting adipose tissue and metabolic activities of visceral fat connected to the release of pro-inflammatory molecules are identified as the main mechanisms involved in obesity and GERD-related symptoms [41]. Based on scientific evidence, obesity plays a role in the development of esophageal adenocarcinoma [42, 43]. Thus, weight management reduces the symptoms of reflux and its complications.

Here, the odds of GERD in depressed individuals were 46% higher than non-depressed. Previous studies indicate a connection between reflux and anxiety disorders and depression [44, 45]. A prospective cohort study reported high levels of anxiety and depression among GERD patients [46]. You et al. observed a significantly higher prevalence of anxiety and depression in the GERD group compared to the control group through the Taiwan National Health Insurance research database [47]. A study with Mendelian randomization method showed a causal and two-way relationship between GERD and increased risk of anxiety disorders and depression [48]. Therefore, clinical specialists in the treatment of GERD patients should focus on the symptoms related to depression and mental disorders in the individual.

## Conclusion

The results indicate that several factors contribute to the risk of GERD, including low physical activity, high consumption of sweets and desserts, low fiber consumption, smoking, alcohol, depression, visceral fat, and central obesity. To prevent GERD and its complications, lifestyle should be improved by following a healthy diet, engaging in regular physical activity, and controlling weight. Finally, clinical specialists should consider the symptoms related to depression in the treatment of GERD patients.

## Data Availability

The data analyzed in the study are available from the corresponding author upon reasonable request.

## Abbreviations

GERD: Gastroesophageal reflux disease
RaNCD: Ravansar non-communicable diseases
WHR: Waist hip ratio
VFA: Visceral fat area
LES: Lower esophageal sphincter
PERSIAN: Prospective Epidemiological Research Studies in Iran
SES: Socio-economic status
PCA: Principal Component analysis
FFQ: Food frequency questionnaire
BMI: Body mass index
PBF: Percent body fat
BFM: Body fat mass
MetS: Metabolic syndrome
IDF: International Diabetes Federation
OR: Odds ratio
CI: Confidence interval
RR: Relative risk
